# Editorial: Addressing the impact of urbanization on health and well-being in African and Asian cities

**DOI:** 10.3389/fpubh.2023.1193519

**Published:** 2023-06-29

**Authors:** Adewale L. Oyeyemi, Ruth Mabry, Lucy-Joy Wachira, Alexandra Gomes, Gustavo De Siqueira

**Affiliations:** ^1^College of Health Solutions, Arizona State University, Phoenix, AZ, United States; ^2^Global Health Consultant, Muscat, Oman; ^3^Department of Physical Education, Exercise and Sports Science, Kenyatta University, Nairobi, Kenya; ^4^LSE Cities, London School of Economics and Political Science, London, United Kingdom; ^5^Department of Urban Planning and Architectural Design, German University of Technology in Oman, Halban, Oman

**Keywords:** urbanization, non-communicable diseases, chronic disease, pollution, physical activity, lifestyles behaviors, Africa, Asia

The urban population of the world will increase from 4.2 billion to approximately 7 billion worldwide by 2050 (1). Most of these growths, up to 90%, will occur in cities of developing countries in Asia and Africa (1). While urbanization brings development and progress to cities by providing access to employment, opportunities, and resources, it could also foster unhealthy lifestyle behaviors, increase exposure to environmental stressors like traffic congestion, air pollution, and worsening inequities in access to infrastructure and resources (2). The unintended consequences of urbanization on planetary and human health are however quite different in different geographies, with higher challenges in in developing countries where the resources to address the burden of diseases and environmental disasters are weak (3, 4). Although urbanization pattern is different in African and Asian cities, issues as rising epidemic of non-communicable diseases like cardiovascular diseases, cancers, chronic respiratory diseases and diabetes that are driven by changes to the physical and social environments brought about by urbanization are common in both regions (5, 6). Most of these health conditions are driven by modifiable behavioral lifestyle factors such as physical inactivity, unhealthy diets, alcohol and tobacco use (7).

Previous studies have highlighted the link between urbanization, health conditions and lifestyle behavioral factors (8, 9). There is also promising evidence of the positive impact of health-friendly urban environments designed to improve lifestyle behavior, health and wellbeing (2, 10). Yet, most of the research on this topic is from high-income countries, predominantly focusing on European and North American cities. Therefore, further investigations to shape this research agenda in the developing countries are needed.

The present Research Topic aimed at widening the knowledge of the role of urban-built environments in improving health and wellbeing in African and Asian cities. The Research Topic brings together three original research, one review, one perspective and one protocol that address critical issues on urbanization and health in Africa and Asia. The contributions explored diverse situations that include China, Ghana, India, Japan, Kenya, and Oman.

Though the broad focus of the contributions is on the impact of urbanization on lifestyle behaviors, pollution, and health and wellbeing, each contribution addresses urbanization and health from unique perspectives. For example, Zhang and Cai explored the impact of new urbanization on environmental pollution in China. The authors utilized an empirical analysis based on spatial modeling of data from 285 prefecture-level cities in China to estimate the environmental pollution control mechanism of the new urbanization development strategies. Their work shows that new urbanization that focuses on improving population mobility, residents' income and consumption, urban and rural areas integration, and urban green coverage can significantly reduce China's environmental pollution in both the long term and the short term.

The work of Tay and Ocansey focused on the impact of urbanization on health and wellbeing in Ghana. Their review of scientific evidence, interventions, and policies shows that environmental risk factors, poor urban planning, water-related problems, unhealthy lifestyle-related behaviors, and socio-economic factors were important urbanization threats to health and wellbeing. They identified government- and individual- level commitment to implementation, surveillance and monitoring of existing policies and interventions as important strategies for mitigating these threats to improve population health and wellbeing in Ghana. As part of a broader natural experimental study to explore approaches to mitigate the impact of urbanization on the overall health and wellbeing of populations in Africa, Wadende et al. conducted stakeholders' perspectives interviews and focus group discussions on the impacts of new and existing Mall and supermarkets in local communities in Kenya. They highlighted how community members have a strong awareness of the negative impact of the rapid change in physical environments on pollution, different types of diseases and health equity. Their findings emphasize the potential utility of health impact assessments for urban interventions to reduce obesogenic environments and improve the health and wellbeing of local populations in Africa.

Koohsari et al. used the lens of interdisciplinary perspective to study the impact of urban design on dog ownership and walking as a means of promoting physical activity behavior in dense urban areas using Japan as a case study. Their findings highlight that key urban built environment designs such as affordable and comfortable buildings, availability and access to public open and green spaces, and activity-friendly mobility infrastructure could positively impact owning and walking dogs. They recommended improvement to these built environmental features as a viable strategy for health and wellbeing promotion in dense urban areas in Japan. To expand this further, De Siqueira et al. explored the role of urban built environments on physical activity and health in Oman. The authors tailored and assessed the physical activity neighborhood environment scale for validity by comparing subjective perceptions of neighborhood environmental features of walkability with objective measures in Muscat, Oman. Their findings highlighted the importance of micro and macro built environmental features including increased access to destinations, easy access to public transport, more places to be active, better infrastructure, and better aesthetic qualities as critical elements of walkability that could be used to improve physical activity, health and wellbeing in Oman.

The last contribution by Sabde et al. described the protocol for a study to evaluate the impact of different housing conditions on the health of under-five year old children in central India. This study provides a rare opportunity to evaluate the health impact of the new national urban renewal mission of the Government of India at a scale among socioeconomically underprivileged urban slum dwellers that are most impacted by ongoing rapid urbanization in India. The outcomes of their research aimed at contributing to guiding future housing-related policy decisions in low- and middle-income countries. Finally, taken together, the six contributions to this Research Topic provide insights into the diversity of methods that have been used to explore the link between the urbanization and health in understudied regions of Africa and Asia. They provide a basis to a small but growing body of context-specific evidence that can guide public health actions to mitigate the worsening impact of urbanization on the health and wellbeing of people in African and Asian cities.

## Author contributions

AO drafted the manuscript and provided approval for publication of content. RM, L-JW, and GD revised the manuscript and provided approval for publication of content. All authors contributed to the article and approved the submitted version.
